# Effectiveness of Integrating HIV Oral Pre-exposure Prophylaxis (PrEP) and Family Planning: A Systematic Review of Initial Implementation Efforts in Low- and Middle-Income Countries

**DOI:** 10.1007/s10461-026-05042-4

**Published:** 2026-01-23

**Authors:** Kevin R. O’Reilly, Ping Teresa Yeh, Virginia Fonner, Caitlin Kennedy, Michael D. Sweat

**Affiliations:** 1 Department of Psychiatry, Division of Global and Community Health, Medical University of South Carolina, Charleston, SC, USA; 2 Department of International Health, Social and Behavioral Interventions Program, Johns Hopkins Bloomberg School of Public Health, Baltimore, MD, USA

**Keywords:** Systematic review, PrEP, Family planning, Integration

## Abstract

Oral pre-exposure prophylaxis (PrEP) and family planning (FP) share key characteristics. Both are preventive, both have high efficacy but effectiveness which can be diminished by inconsistent use and both are particularly beneficial for adolescent girls and young women (AGYW) in certain lower- and middle-income countries. When the efficacy of PrEP was proven in field trials, an effort to implement PrEP programs for the populations that would most benefit from it was launched. For AGYW, making PrEP available through existing FP services was seen as a natural opportunity for integration and was the primary effort and until recently, only experience in implementation of PrEP for AGYW. In this systematic review, we attempted to discover whether the integrated delivery of PrEP and FP results in the uptake and consistent use of either or both interventions. We found that no studies that met our inclusion criteria as no valid comparison for a PrEP/FP integration was discovered. Nonetheless, valuable information on the feasibility and acceptability of PrEP was gleaned from some of these studies, much less on the impact of PrEP promotion on FP acceptance and use. Providing PrEP to eligible individuals is an urgent public health priority. Doing so while enhancing uptake and use of FP is equally important. Future studies would benefit from a more encompassing view of this important integration.

## Introduction

Pre-exposure prophylaxis (PrEP) has revolutionized HIV prevention. The efficacy of daily oral PrEP in preventing acquisition of HIV was first shown in men who have sex with men in 2010 [1] and soon after in heterosexual couples in East Africa in 2012 [2]. The World Health Organization (WHO) recommended daily oral PrEP as an HIV prevention option in 2015 and has subsequently recommended additional PrEP modalities, including the dapivirine vaginal ring in 2021, injectable cabotegravir in 2022, and injectable lenacapavir in 2025 [3]. The potential impact of PrEP is large, especially among adolescent girls and young women (AGYW), for whom the risk of HIV infection remains high, particularly in eastern and southern Africa [4]. Gender inequality has been found to be a key driver in predominantly heterosexual generalized HIV epidemics, as AGYW in such settings may have limited access to care as well as limited agency to take the actions needed to reduce their risks or address their health needs [5]. Providing PrEP to this priority population has thus become an important focus of HIV prevention efforts. The DREAMS Partnership is an example of this concerted effort [6], though the recent dramatic cuts in funding from the United States and other high-income governments have so far curtailed many of these efforts.

Following the demonstration of the efficacy of PrEP, efforts to provide access where sexually active AGYW population received sexual and reproductive health care were launched. Given that family planning (FP) services for AGYW are well established and have been promoted for more than sixty years, the logic of integrating the two for increased PrEP availability was compelling. The opportunity to prevent HIV acquisitions, detect HIV infections early in AGYW, to access HIV treatment and reduce the risk of mother-to-child transmission are all potential benefits of integrating PrEP and its attendant HIV services into FP services. Increased FP usage and the reduction of unintended pregnancies that could result from this type of integration would also address another longstanding public health priority.

Integration has received increased attention in health services research in low- and middle-income countries (LMICs). It holds the promise to increase coverage and efficiency, while decreasing costs of service delivery [7]. An interagency working group of IPPF, UNFPA and WHO defined integration of HIV and sexual and reproductive health (SRH) as combining different types of services to maximize outcomes, adding that it can be bi-directional, with SRH services integrated into HIV services and HIV services integrated into SRH services [8]. A recent scoping review has shown that integration of PrEP and FP is an increasingly common strategy [9]. However, whether this strategy is effective at delivering PrEP, and what effects on FP acceptance and use may result, was not clear in that review. In this systematic review, we attempt to answer whether the integrated delivery of PrEP and FP result in the uptake and consistent use of both interventions.

## Objective

To conduct a systematic review of the effectiveness of integrating PrEP delivery with family planning services in LMICs for both PrEP and FP uptake.

## Methods

This review was conducted as part of The Evidence Project, which uses standard procedures to assess the evidence for effectiveness of behavioral and structural interventions related to HIV prevention in LMICs [10]. We follow standard systematic review methods and present findings following the Preferred Reporting Items for Systematic Reviews and Meta-Analyses (PRISMA) statement [11].

To qualify for inclusion in our review, articles needed to satisfy the following criteria:

1. Published between January 2012 (the year the first PrEP trials in LMICs were published) and the search date of 30 May 2025.
2. Conducted in LMICs, as defined by the World Bank country classifications [12].
3. Population: Adult or adolescent women who are HIV negative or HIV serostatus unknown.
4. Evaluated the integration of PrEP services and family planning services.
  - Getting integrated diagnostic or treatment services to clients at the site of service delivery - this could include co-location of services, as well as screening coupled with referral (passive or facilitated).
  - WHO has defined integration as the ‘management and delivery of health services so that clients receive a continuum of preventive and curative services, according to their needs over time and across different levels of the health system’ [13]. For the purposes of this review, we used this definition of integration and focus on the service delivery level, rather than at the policy or other levels.
  - We anticipated mostly studies that integrate PrEP into existing FP services but also included studies that integrate FP into existing PrEP services, or that start providing both services simultaneously.
5. Study design provides a quantitative comparison between those who were exposed to FP-PrEP service integration to those who were not exposed, whether pre-post (e.g. before integration vs. after integration) or multi-arm (site with integration vs. site without/with a different form of integration). This could include randomized controlled trials (RCTs), non-randomized trials, and comparative observational studies.
6. Report at least one quantitative pre-post or multi-arm comparison for outcomes of interest:
  - PrEP related outcomes: PrEP referral, PrEP uptake (proportion of participants using PrEP), consistency of PrEP use, duration of PrEP use, HIV incidence.
  - FP related outcomes: uptake, continuation, unmet need.

We employed no restrictions by population subgroup or language of publication.

We searched PubMed, CINAHL, PsycINFO, Sociological Abstracts and EMBASE using the search terms drawn from three key concepts: HIV, PrEP and FP (Appendix). Search results were screened in a multi-stage process, first by a single reviewer reviewing titles and abstracts to exclude citations that clearly did not meet the inclusion criteria, and then by two reviewers in duplicate reviewing titles and abstracts to determine likely eligibility, then by reviewing full text articles in duplicate. Differences were resolved through discussion and consensus.

Data were extracted independently by two reviewers using standardized forms, and any coding differences were resolved through discussion and consensus. Data were extracted on sixteen content areas: (1) citation information, (2) study inclusion criteria, (3) study methods, (4) study population characteristics, (5) setting, (6) sampling, (7) study design, (8) unit of analysis, (9) loss to follow up rates, (10) study group characteristics, (11) intervention characteristics, (12) intervention topic-specific questions, (13) outcome measures, (14) eligible outcome results, (15) risk of bias and (16) additional information such as costs, limitations, community acceptance, etc.

## Results

**Fig. 1:**
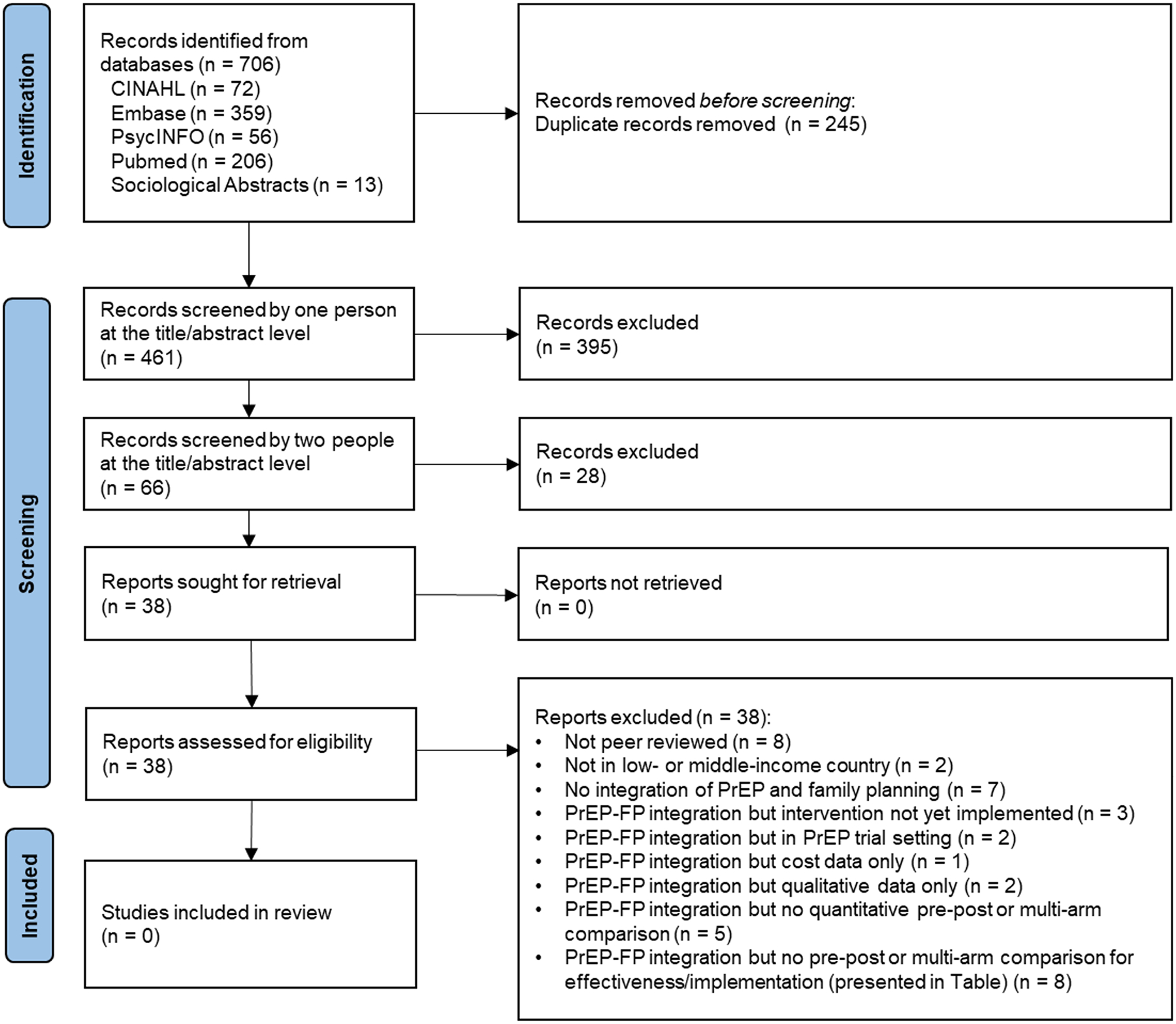
PRISMA flowchart of steps in systematic review of PrEP and FP integration

The database search yielded 706 unique citations (Fig. 1). After removing duplicates, 461 articles remained for initial screening, and 66 articles were screened in duplicate. Further exclusions left 38 articles for full text review. When this was concluded, eight articles initially appeared eligible for inclusion in this review. However, after addressing issues relating to integration and valid comparisons addressing effectiveness of that integration that were essential elements of our inclusion criteria, none of the eight studies fully qualified for inclusion.

While many relevant studies were excluded for not meeting the inclusion criteria, some provided helpful information on acceptability and feasibility, important when considering how integration of PrEP with FP has occurred and been studied. Of the initial eight studies, five offered PrEP and contraception in the context of FP services [14–18]. All took place in east and southern Africa and focused on AGYW. These five studies primarily assessed the acceptability and feasibility of offering the interventions in an integrated manner. Their findings are presented in Table 1.

Four studies addressed the acceptance of PrEP when offered in the context of FP services. In Kenya and South Africa, 94% of the 2550 eligible women offered PrEP in FP clinics initiated its use [14]. In a study of nine South African sites that participated in the ECHO trial, which provided both PrEP and family planning, PrEP use was reported by 26% of eligible women at follow-up [15]. In Kenya, following newly adopted national guidelines for PrEP, a feasibility study found that 22% of women overall, and more than 90% of women with at least one risk factor for HIV, accepted PrEP when offered in reproductive health clinics [16]. Another study in Kenya reviewed 6877 medical records of AGYW receiving post abortion care: 57% had been offered PrEP and 14.1% had initiated PrEP use [17].

One South African study employed the opposite approach and assessed the promotion of FP for women seeking or using PrEP [18]. This study found that 62.3% of PrEP initiators were current FP users. Among those not using family planning, 32.3% elected a method at PrEP initiation, increasing overall contraceptive prevalence by 12.2% [18].

Continuation on PrEP was also measured in two studies. In Kenya and South Africa, only 749 women or 31% of the 2397 AGYW who initiated PrEP returned after one month for a refill of the product [14]. Of those who could be assessed at the six-month follow-up, 20% (128 out of 646) continued with PrEP over that time without a gap of 15 days or more in PrEP refills. An additional 92 (14% of 646) restarted PrEP after such a gap. HIV incidence was 2.2 per 100 person years (95% CI 1.2–3.5) in AGYW who did not have PrEP dispensed in the study period prior to seroconversion or had serum TFV-DP levels that suggested less than 4 doses per week used in the six weeks prior to seroconversion [14]. A South African study using the ECHO cohort assessed PrEP adherence by tracking HIV incidence, which decreased from 4.65 per 100 person-years before PrEP introduction to 2.16 after introduction (adjusted incidence rate ratio 0.45, 95% CI 0.25–0.82, *p* = 0.0085) [15].

Three other studies addressed variations on the theme of integrated FP/PrEP services. In Kenya, nurses positioned in pharmacies intercepted AGYW purchasing contraception, primarily emergency contraception, and offered the initial one-month supply of PrEP, following national guidelines [19]. Overall, PrEP uptake in this small study was 85%; at one month, 82% had initiated PrEP use, and 68% of these intended to continue use. In South Africa, a pilot cluster randomized controlled trial offered both PrEP and FP in hair salons: nearly half of the 134 participants in the intervention salons accepted salon-based PrEP, and 89% accepted salon-based contraception [20]. Finally, a study in Zambia promoted community-based delivery of SRH services, including referral for PrEP, but found no impact on PrEP use [21].

## Discussion

Our review was designed to discover and review evidence of the effectiveness of integrating PrEP provision and support alongside FP into existing sexual and reproductive health services. These studies clearly presented important and, in most cases, encouraging findings on the acceptability of offering PrEP in the context of FP services. Given the priority for preventing HIV infection in AGYW, especially in high-burden African settings, this evidence is important. Contact with antenatal care has been demonstrated to be a key avenue for women to learn their HIV status for the first time, though service gaps often result in missed opportunities [22]. Integrating HIV testing into FP service delivery has been less commonly undertaken [23], though this integration has shown potential to reach AGYW who may lack the awareness or the agency to seek HIV testing on their own [24].

Given this clear need, we expected to find multiple studies that focused on the integration of PrEP and FP services meeting our inclusion criteria. The relative novelty of PrEP as an intervention may suggest that our review was premature. It is equally possible that our focus on assessing the effectiveness of integration in a bidirectional manner may not be widely shared. While both HIV and FP are longstanding health goals, the difference in the level of support provided through vertical funding streams does not suggest equal priority for the two: in 2023, the United States funded HIV programs in the LMICs at nearly 12 times the funding it provided for family planning and reproductive health programs [25, 26].

We did find evidence of the feasibility and acceptability of providing PrEP in FP clinics. Coupled with the strong evidence of the efficacy of oral PrEP as an intervention to prevent acquisition of HIV infection [27], this is encouraging. Future work on this topic may well be best addressed through further implementation science, in an effort to discover effective ways to jointly deliver both interventions, for the benefit of the intended outcomes of both.

The bidirectional effectiveness of this integrated approach, however, could not be adequately assessed in this review. To be included in our review, studies needed to present multi-arm or pre/post comparisons of the integration of the two services. While FP is a well-established intervention with decades of evidence and experience in its implementation to support it, PrEP is relatively new. As a result, studies conducted prior to the cutoff date for our review could not provide a suitable comparator of other means of implementation to assess the effectiveness of an integrated implementation in the context of FP services. Neither “pre” data for PrEP effectiveness prior to its integration into FP clinics nor multi arm studies comparing this integration to other means of providing PrEP were found. A scan of the literature beyond our focus on LMICs did not reveal additional attention to integrated PrEP/FP delivery in high income countries, but an emphasis on the barriers and facilitators of PrEP uptake [28].

The evidence presented in these studies primarily focused on PrEP acceptance, use and continuation. Only one study measured the impact on family planning; it found that PrEP introduction could also stimulate acceptance of contraception among PrEP initiators who were not previously using FP [18]. Further studies focusing on the impact of PrEP integration on FP use are warranted.

Integration has been defined as the ‘management and delivery of health services so that clients receive a continuum of preventive and curative services, according to their needs over time and across different levels of the health system’ [13]. Integration of HIV and SRH faces numerous challenges, however, including vertical and often unequal funding streams which function parallel to, not as an integrated element of, national health care delivery [29]. In short, the elements to be integrated, which would clearly benefit AGYW in need, have been largely funded by external donors, not by national programs. Since the beginning of 2025, much of that funding has been halted, through the destruction of USAID and the limitation of PEPFAR-sponsored activities, or reduced as other key donors have followed the lead of the United States and reduced their contributions for foreign assistance. Since that time, national programs have been struggling to fill the gaps created, a challenging task when not given adequate warning to plan for such a change.

More relevant for this review, a scoping review of the topic defined integration as combining different types of services to maximise outcomes and can be bi-directional, with sexual and reproductive health (SRH) services integrated into HIV services and HIV services integrated into SRH services [9]. Integration of HIV prevention interventions within existing SRH services can improve both access and effectiveness of FP care [8]. The adoption of this definition for PrEP and FP integration would allow for a more robust assessment of the effectiveness of that integration, by addressing additional questions such as: *Has the integration of PrEP delivery in FP clinics attracted new users? Has it facilitated continuation of FP use? Have additional resources (funding*, *training expanded facilities*, *etc.) been made available to the integrated clinics to adjust to the additional demand on staff time and facilities?* The literature we reviewed suggests that the goal is primarily the expansion of PrEP availability and its uptake and continued use. An awareness of the potential impact of that integration, both positive and negative, on the FP services being provided would present a more complete picture, one that reflects the increasing importance being accorded to integration of services and universal health care. Integrating PrEP and FP to be delivered jointly in external sites where neither service had been previously available, for example, may offer the potential to increase uptake and coverage of both interventions. Under the rubric of universal health care, this type of expansion may have merit. Some studies are beginning to explore less conventional approaches to providing PrEP and contraception, such as the studies documenting PrEP implementation in pharmacies for women seeking contraception [19] or the promotion of both PrEP and FP through hair salons [20]. Studies like these offer more diverse mechanisms for implementing both interventions and potentially meaningful comparisons to the approaches described in the articles we describe above.

Studies of the integration of PrEP and contraception in FP clinical settings could also be enriched by examining implementation issues. Until the recent cut-off of U.S. support, many integration efforts were driven by HIV funding and actors. Feasibility, acceptability, and cost implications experienced by FP providers, important implementation issues, were less explored. Costing the time and work burden PrEP provision integrated into existing FP services places on FP provision is necessary before an assessment of the effectiveness of this integration can be made. Additionally, as options for PrEP expand, it will be important to understand barriers and facilitators to integrating FP and PrEP in the context of having more than one PrEP modality available, given both the potential complexity of counselling women on multiple methods, as well as potential synergies between modality and visit schedules for newer PrEP products (e.g., cabotegravir, lenacapavir). Anticipating and planning for the potential challenges posed by this integrated implementation, considering the different schedules of clinic visits for refills, resupply or reinjection of the two interventions is also crucial.

There are significant limitations to this systematic review. The lack of available publications that address our central question has created a challenge for the presentation of the results obtained. This can also be viewed as a strength, however, in that our review has highlighted that an important question of integrated services has not yet been addressed. Identifying this gap in the research record may well stimulate efforts to fill it. It is also possible that our emphasis on a bidirectional effect of integration, with emphasis on the impact of PrEP on FP services and acceptance, may be seen as a lesser priority given the challenge on implementing PrEP on a wide scale for the first time. What is likely to transpire now in these integration efforts with the widescale disruption of U.S. support for both HIV prevention programs and for FP is unclear. New opportunities for meaningful prevention through the use of lenacapavir, recently approved by the U.S. FDA and endorsed by WHO, and future products under development should be encouraging for meaningful HIV prevention but implementation has now become more challenging. This review only identified studies related to integrating oral PrEP and family planning; studies did not examine integration of FP services with other PrEP modalities. More research will be needed to understand both the effectiveness and implementation of integrating FP and PrEP in the context of a multi-method PrEP market.

## Conclusion

We undertook a systematic review of the effectiveness of integrating PrEP provision with FP in sexual and reproductive health clinical settings. While multiple studies demonstrate the acceptability of this approach, we identified no studies which evaluated effectiveness of integration using a comparative design. Providing PrEP to eligible individuals is an urgent public health priority. Doing so while enhancing uptake and use of FP is equally important. Future studies would benefit from a more encompassing view of this important integration.

## Supplementary Material

Appendix Search Strategy

**Supplementary Information** The online version contains supplementary material available at https://doi.org/10.1007/s10461-026-05042-4.

## Figures and Tables

**Table 1:** Studies addressing integration of PrEP and FP in SRH services: assessing feasibility and acceptability of PrEP implementation

Author year	Population and setting	Intervention	Study design	Primary outcomes	Effect on PrEP initiation and persistence	Effects on FP uptake and persistence
Celum et al. 2022 [14]	AGYW Kisumu, Kenya Cape Town, Johannesburg, South Africa	PrEP offered in facility-based clinics and mobile van. recruitment *through peer outreach, social media and clinics*, counseled on HIV risk reduction, offered PrEP, STI testing and treatment, HIV testing, and contraception	Prospective implementation science evaluation comparing PrEP persisters to non-persisters Compares PrEP users to non-users post intervention	PrEP initiation, use, persistence	94% initiated, 31% of those refilled at 1 month follow up visit. At 6 month follow up, 20% persisted on PrEP (128/646); 14% (92/646) had a gap but restarted. HIV incidence in PrEP users: 2.2 per 100 person-years	Not reported
Donnell et al., 2021 [15]	Women aged 16–35 9 of 12 South African ECHO trial sites	Standard HIV prevention package at all visits: testing, risk reduction counseling, info about PrEP, condom provision and STI syndromic management	Nested interrupted time series at 9 South African sites w/on-site access to PrEP	HIV incidence before vs. after PrEP implementation.	2124 women followed after PrEP implementation; 543 (26%) reported PrEP use; 12 seroconversions (incidence 2.16) in 556 person-years	Not reported
Mugwanya et al., 2019 [16]	AGYW Kisumu, Kenya	PrEP delivered in MCH and FP clinics to AGYW at substantial HIV risk	Stepped wedge cluster randomized trial to evaluate implementation of national guidelines for PrEP	Compares PrEP initiation by use and by type of FP used; PrEP initiation by demographic and risk factors	22% accepted PrEP, with acceptance > 90% in women with at least one risk factor for HIV; 39% not on FP, 24% with implant and 15% on injectable. 41% of PrEP initiators returned for refill	Not reported
Zia et al., 2024 [17]	AGYW (post abortion care patients) 14 clinics offering services for PAC in Kisumu, Nairobi, Thika	PrEP integrated into PAC services	“implementation science driven evaluation” to evaluate uptake and feasibility Modified Poisson regression	Uptake of PrEP by women post-abortion	Of 6877 case records reviewed, PrEP offered to 57.4%; 14,1% initiated; consistent supply of PrEP was important influence on acceptance and uptake	
Pleaner, et al. 2021 [18]	AGYW 8 health care facilities and 4 mobile clinics in the South African provinces	Analysis of baseline routine monitoring data to examine contraceptive use and uptake in AGYW accessing PrEP in project sites; improving M&E data to capture bidirectional nature of integration	Implementation science/observational study Compares Contraceptive users vs. non-users; method chosen by non-users at PrEP initiation	Contraceptive use and uptake in AGYW accessing PrEP in project sites	N/A (all participants were PrEP users)	32.3% non contraceptors accepted a method at PrEP initiation; method uptake at PrEP initiation increased overall contraceptive prevalence by 12.2 to 74.5%

*AGYW* adolescent girls and young women, *M&E* monitoring and evaluation, *MCH* maternal child health, *N/A* not applicable, *PAC* post abortion care, *PrEP* pre-exposure prophylaxis, *STI* sexually transmitted infection

## Data Availability

Available upon request to the corresponding author.
