# Supplementary material for: Effectiveness of Integrating HIV Oral Pre-exposure Prophylaxis (PrEP) and Family Planning: A Systematic Review of Initial Implementation Efforts in Low- and Middle-Income Countries
Source: AIDS Behav. 2026 Jan 23;30(7):2166–73. doi: 10.1007/s10461-026-05042-4 (PMC13244559; doi:10.1007/s10461-026-05042-4)
Supplement: Supplementary file 1 — Supplementary Material 1 [file 10461_2026_5042_MOESM1_ESM.docx]

**Appendix 1**

**Search strategy**

The following electronic databases will be searched: PubMed, PsycINFO, CINAHL (Cumulative Index to Nursing and Allied Health Literature), Embase, and Sociological Abstracts using a combination of search terms below. Secondary reference searching will also be conducted on all studies included in the review.

**Search terms:**

**3 concepts: HIV, PrEP, and Family Planning**

**Pubmed:** (HIV[Title/Abstract] or HIV[MeSH]) AND (pre-exposure prophylaxis [MeSH] OR "preexposure prophylaxis"[Title/Abstract] OR "pre-exposure prophylaxis"[Title/Abstract] OR "pre exposure prophylaxis"[Title/Abstract] OR PrEP[Title/Abstract]) AND (“Family Planning services” [MeSH] OR “Family Planning service” [Title/Abstract] OR “Family Planning” [Title/Abstract] OR “Planned Pregnancy” [Title/Abstract] OR “Planned Pregnancies” [Title/Abstract] OR “Family Planning Programs” [Title/Abstract] OR “Family Planning Program” [Title/Abstract] OR “Family Planning Programmes” [Title/Abstract] OR “Family Planning Programme” [Title/Abstract] OR “Reproductive counselling” [Title/Abstract] OR “Birth Spacing” [Title/Abstract] OR “Birth Control” [Title/Abstract] OR “Contraception” [MeSH] OR “Fertility control” [Title/Abstract] OR “family planning center” [Title/Abstract] OR “family planning centers” [Title/Abstract] OR “family planning centre” [Title/Abstract] OR “family planning centres” [Title/Abstract] OR “Family planning clinic”[Title/Abstract] OR “Family Planning Clinics” [Title/Abstract] OR “Abortion clinic” [Title/Abstract] OR “Abortion clinics” [Title/Abstract] OR “Abortion center” [Title/Abstract] OR “Abortion centers” [Title/Abstract] OR “Abortion centre” [Title/Abstract] OR “Abortion centres” [Title/Abstract] OR “Contraceptive services” [Title/Abstract] OR “Reproductive health services” [mesh] OR “Reproductive health service” [Title/Abstract] OR "Maternal health services” [Title/Abstract] OR “Sexual and Reproductive health service” [Title/Abstract] OR “Sexual and Reproductive health services” [Title/Abstract] OR “Fertility control clinics” [Title/Abstract] OR “Fertility control clinic” [Title/Abstract] OR “Fertility control center” [Title/Abstract] OR “Fertility control centers” [Title/Abstract] OR “Fertility control centre” [Title/Abstract] OR “Fertility control centres” [Title/Abstract] OR “Fertility control services” [Title/Abstract])

**CINAHL, PsycINFO:** (MH HIV OR AB HIV OR TI HIV) AND (MH "pre-exposure prophylaxis" OR AB "preexposure prophylaxis” OR AB "pre-exposure prophylaxis" OR AB "pre exposure prophylaxis" OR AB PrEP OR TI "preexposure prophylaxis” OR TI "pre-exposure prophylaxis" OR TI "pre exposure prophylaxis" OR TI PrEP) AND AND (MH “Family Planning services” OR AB “Family Planning service” OR AB “Family Planning” OR AB “Planned Pregnancy” OR AB “Planned Pregnancies” OR AB “Family Planning Programs” OR AB “Family Planning Program” OR AB “Family Planning Programmes” OR AB “Family Planning Programme” OR AB “Reproductive counselling” OR AB “Birth Spacing” OR AB “Birth Control” OR MH “Contraception” OR “Fertility control” OR AB “family planning center” OR AB “family planning centers” OR AB “family planning centre” OR AB “family planning centres” OR AB “Family planning clinic”OR AB “Family Planning Clinics” OR AB “Abortion clinic” OR AB “Abortion clinics” OR AB “Abortion center” OR AB “Abortion centers” OR AB “Abortion centre” OR AB “Abortion centres” OR AB “Contraceptive services” OR MH “Reproductive health services” OR “Reproductive health service” OR AB "Maternal health services” OR AB “Sexual and Reproductive health service” OR AB “Sexual and Reproductive health services” OR AB “Fertility control clinics” OR AB “Fertility control clinic” OR AB “Fertility control center” OR AB “Fertility control centers” OR AB “Fertility control centre” OR AB “Fertility control centres” OR AB “Fertility control services” OR TI “Family Planning services” OR TI “Family Planning service” OR TI “Family Planning” OR TI “Planned Pregnancy” OR TI “Planned Pregnancies” OR TI “Family Planning Programs” OR TI “Family Planning Program” OR TI “Family Planning Programmes” OR TI “Family Planning Programme” OR TI “Reproductive counselling” OR TI “Birth Spacing” OR TI “Birth Control” OR TI “Contraception” OR “Fertility control” OR TI “family planning center” OR TI “family planning centers” OR TI “family planning centre” OR TI “family planning centres” OR TI “Family planning clinic” OR TI “Family Planning Clinics” OR TI “Abortion clinic” OR TI “Abortion clinics” OR TI “Abortion center” OR TI “Abortion centers” OR TI “Abortion centre” OR TI “Abortion centres” OR TI “Contraceptive services” OR TI “Reproductive health services” OR “Reproductive health service” OR TI "Maternal health services” OR TI “Sexual and Reproductive health service” OR TI “Sexual and Reproductive health services” OR TI “Fertility control clinics” OR TI “Fertility control clinic” OR TI “Fertility control center” OR TI “Fertility control centers” OR TI “Fertility control centre” OR TI “Fertility control centres” OR TI “Fertility control services”)

**Sociological Abstracts**

noft(("Family Planning services" OR "Family Planning service" OR "Family Planning" OR "Planned Pregnancy" OR "Planned Pregnancies" OR "Family Planning Programs" OR "Family Planning Program" OR "Family Planning Programmes" OR "Family Planning Programme" OR "Reproductive counselling" OR "Birth Spacing" OR "Birth Control" OR "Contraception" OR "Fertility control" OR "family planning center" OR "family planning centers" OR "family planning centre" OR "family planning centres" OR "Family planning clinic" OR "Family Planning Clinics" OR "Abortion clinic" OR "Abortion clinics" OR "Abortion center" OR "Abortion centers" OR "Abortion centre" OR "Abortion centres" OR "Contraceptive services" OR "Reproductive health services" OR "Reproductive health service" OR "Maternal health services" OR "Sexual and Reproductive health service" OR "Sexual and Reproductive health services" OR "Fertility control clinics" OR "Fertility control clinic" OR "Fertility control center" OR "Fertility control centers" OR "Fertility control centre" OR "Fertility control centres" OR "Fertility control services")) AND noft(HIV) AND noft((pre-exposure prophylaxis OR "preexposure prophylaxis" OR "pre-exposure prophylaxis" OR "pre exposure prophylaxis" OR PrEP)) AND pd(>20120101)

**EMBASE**

(('preexposure prophylaxis':ab,ti OR 'pre-exposure prophylaxis':ab,ti OR 'pre exposure prophylaxis':ab,ti OR prep:ab,ti) AND hiv:ab,ti AND (‘Family Planning services’:ab,ti OR ‘Family Planning service’:ab,ti OR ‘Family Planning’:ab,ti OR ‘Planned Pregnancy’:ab,ti OR ‘Planned Pregnancies’:ab,ti OR ‘Family Planning Programs’:ab,ti OR ‘Family Planning Program’:ab,ti OR ‘Family Planning Programmes’:ab,ti OR ‘Family Planning Programme’:ab,ti OR ‘Reproductive counselling’:ab,ti OR ‘Birth Spacing’:ab,ti OR ‘Birth Control’:ab,ti OR ‘Contraception’:ab,ti OR ‘Fertility control’:ab,ti OR ‘family planning center’:ab,ti OR ‘family planning centers’:ab,ti OR ‘family planning centre’:ab,ti OR ‘family planning centres’:ab,ti OR ‘Family planning clinic’:ab,ti OR ‘Family Planning Clinics’:ab,ti OR ‘Abortion clinic’:ab,ti OR ‘Abortion clinics’:ab,ti OR ‘Abortion center’:ab,ti OR ‘Abortion centers’:ab,ti OR ‘Abortion centre’:ab,ti OR ‘Abortion centres’:ab,ti OR ‘Contraceptive services’:ab,ti OR ‘Reproductive health services’:ab,ti OR ‘Reproductive health service’:ab,ti OR ‘Maternal health services’:ab,ti OR ‘Sexual and Reproductive health service’:ab,ti OR ‘Sexual and Reproductive health services’:ab,ti OR ‘Fertility control clinics’:ab,ti OR ‘Fertility control clinic’:ab,ti OR ‘Fertility control center’:ab,ti OR ‘Fertility control centers’:ab,ti OR ‘Fertility control centre’:ab,ti OR ‘Fertility control centres’:ab,ti OR ‘Fertility control services’:ab,ti))

**Search Period:** 2012 through 5 May 2023 updated 13 Jun 2024 updated 19 Nov 2024

| **Database** | **Search Terms (including filters/limits)** | **Search date** | **Results** |
| --- | --- | --- | --- |
| Pubmed | ((HIV[Title/Abstract] or HIV[MeSH]) AND (pre-exposure prophylaxis [MeSH] OR "preexposure prophylaxis"[Title/Abstract] OR "pre-exposure prophylaxis"[Title/Abstract] OR "pre exposure prophylaxis"[Title/Abstract] OR PrEP[Title/Abstract]) AND ("Family Planning services" [MeSH] OR "Family Planning service" [Title/Abstract] OR "Family Planning" [Title/Abstract] OR "Planned Pregnancy" [Title/Abstract] OR "Planned Pregnancies" [Title/Abstract] OR "Family Planning Programs" [Title/Abstract] OR "Family Planning Program" [Title/Abstract] OR "Family Planning Programmes" [Title/Abstract] OR "Family Planning Programme" [Title/Abstract] OR "Reproductive counselling" [Title/Abstract] OR "Birth Spacing" [Title/Abstract] OR "Birth Control" [Title/Abstract] OR "Contraception" [MeSH] OR "Fertility control" [Title/Abstract] OR "family planning center" [Title/Abstract] OR "family planning centers" [Title/Abstract] OR "family planning centre" [Title/Abstract] OR "family planning centres" [Title/Abstract] OR "Family planning clinic"[Title/Abstract] OR "Family Planning Clinics" [Title/Abstract] OR "Abortion clinic" [Title/Abstract] OR "Abortion clinics" [Title/Abstract] OR "Abortion center" [Title/Abstract] OR "Abortion centers" [Title/Abstract] OR "Abortion centre" [Title/Abstract] OR "Abortion centres" [Title/Abstract] OR "Contraceptive services" [Title/Abstract] OR "Reproductive health services" [mesh] OR "Reproductive health service" [Title/Abstract] OR "Maternal health services" [Title/Abstract] OR "Sexual and Reproductive health service" [Title/Abstract] OR "Sexual and Reproductive health services" [Title/Abstract] OR "Fertility control clinics" [Title/Abstract] OR "Fertility control clinic" [Title/Abstract] OR "Fertility control center" [Title/Abstract] OR "Fertility control centers" [Title/Abstract] OR "Fertility control centre" [Title/Abstract] OR "Fertility control centres" [Title/Abstract] OR "Fertility control services" [Title/Abstract])) AND (("2012/01/01"[Date - Publication] : "3000"[Date - Publication])) | 5 May 2023 | 152 |
| PsycINFO | "(MH HIV OR AB HIV OR TI HIV) AND (MH "pre-exposure prophylaxis" OR AB "preexposure prophylaxis” OR AB "pre-exposure prophylaxis" OR AB "pre exposure prophylaxis" OR AB PrEP OR TI "preexposure prophylaxis” OR TI "pre-exposure prophylaxis" OR TI "pre exposure prophylaxis" OR TI PrEP) AND AND (MH “Family Planning services” OR AB “Family Planning service” OR AB “Family Planning” OR AB “Planned Pregnancy” OR AB “Planned Pregnancies” OR AB “Family Planning Programs” OR AB “Family Planning Program” OR AB “Family Planning Programmes” OR AB “Family Planning Programme” OR AB “Reproductive counselling” OR AB “Birth Spacing” OR AB “Birth Control” OR MH “Contraception” OR “Fertility control” OR AB “family planning center” OR AB “family planning centers” OR AB “family planning centre” OR AB “family planning centres” OR AB “Family planning clinic”OR AB “Family Planning Clinics” OR AB “Abortion clinic” OR AB “Abortion clinics” OR AB “Abortion center” OR AB “Abortion centers” OR AB “Abortion centre” OR AB “Abortion centres” OR AB “Contraceptive services” OR MH “Reproductive health services” OR “Reproductive health service” OR AB "Maternal health services” OR AB “Sexual and Reproductive health service” OR AB “Sexual and Reproductive health services” OR AB “Fertility control clinics” OR AB “Fertility control clinic” OR AB “Fertility control center” OR AB “Fertility control centers” OR AB “Fertility control centre” OR AB “Fertility control centres” OR AB “Fertility control services” OR TI “Family Planning services” OR TI “Family Planning service” OR TI “Family Planning” OR TI “Planned Pregnancy” OR TI “Planned Pregnancies” OR TI “Family Planning Programs” OR TI “Family Planning Program” OR TI “Family Planning Programmes” OR TI “Family Planning Programme” OR TI “Reproductive counselling” OR TI “Birth Spacing” OR TI “Birth Control” OR TI “Contraception” OR “Fertility control” OR TI “family planning center” OR TI “family planning centers” OR TI “family planning centre” OR TI “family planning centres” OR TI “Family planning clinic” OR TI “Family Planning Clinics” OR TI “Abortion clinic” OR TI “Abortion clinics” OR TI “Abortion center” OR TI “Abortion centers” OR TI “Abortion centre” OR TI “Abortion centres” OR TI “Contraceptive services” OR TI “Reproductive health services” OR “Reproductive health service” OR TI "Maternal health services” OR TI “Sexual and Reproductive health service” OR TI “Sexual and Reproductive health services” OR TI “Fertility control clinics” OR TI “Fertility control clinic” OR TI “Fertility control center” OR TI “Fertility control centers” OR TI “Fertility control centre” OR TI “Fertility control centres” OR TI “Fertility control services”) Published Date: 20120101- AND Apply related words; Apply equivalent subjects” | 5 May 2023 | 43 |
| CINAHL | "(MH HIV OR AB HIV OR TI HIV) AND (MH "pre-exposure prophylaxis" OR AB "preexposure prophylaxis” OR AB "pre-exposure prophylaxis" OR AB "pre exposure prophylaxis" OR AB PrEP OR TI "preexposure prophylaxis” OR TI "pre-exposure prophylaxis" OR TI "pre exposure prophylaxis" OR TI PrEP) AND AND (MH “Family Planning services” OR AB “Family Planning service” OR AB “Family Planning” OR AB “Planned Pregnancy” OR AB “Planned Pregnancies” OR AB “Family Planning Programs” OR AB “Family Planning Program” OR AB “Family Planning Programmes” OR AB “Family Planning Programme” OR AB “Reproductive counselling” OR AB “Birth Spacing” OR AB “Birth Control” OR MH “Contraception” OR “Fertility control” OR AB “family planning center” OR AB “family planning centers” OR AB “family planning centre” OR AB “family planning centres” OR AB “Family planning clinic”OR AB “Family Planning Clinics” OR AB “Abortion clinic” OR AB “Abortion clinics” OR AB “Abortion center” OR AB “Abortion centers” OR AB “Abortion centre” OR AB “Abortion centres” OR AB “Contraceptive services” OR MH “Reproductive health services” OR “Reproductive health service” OR AB "Maternal health services” OR AB “Sexual and Reproductive health service” OR AB “Sexual and Reproductive health services” OR AB “Fertility control clinics” OR AB “Fertility control clinic” OR AB “Fertility control center” OR AB “Fertility control centers” OR AB “Fertility control centre” OR AB “Fertility control centres” OR AB “Fertility control services” OR TI “Family Planning services” OR TI “Family Planning service” OR TI “Family Planning” OR TI “Planned Pregnancy” OR TI “Planned Pregnancies” OR TI “Family Planning Programs” OR TI “Family Planning Program” OR TI “Family Planning Programmes” OR TI “Family Planning Programme” OR TI “Reproductive counselling” OR TI “Birth Spacing” OR TI “Birth Control” OR TI “Contraception” OR “Fertility control” OR TI “family planning center” OR TI “family planning centers” OR TI “family planning centre” OR TI “family planning centres” OR TI “Family planning clinic” OR TI “Family Planning Clinics” OR TI “Abortion clinic” OR TI “Abortion clinics” OR TI “Abortion center” OR TI “Abortion centers” OR TI “Abortion centre” OR TI “Abortion centres” OR TI “Contraceptive services” OR TI “Reproductive health services” OR “Reproductive health service” OR TI "Maternal health services” OR TI “Sexual and Reproductive health service” OR TI “Sexual and Reproductive health services” OR TI “Fertility control clinics” OR TI “Fertility control clinic” OR TI “Fertility control center” OR TI “Fertility control centers” OR TI “Fertility control centre” OR TI “Fertility control centres” OR TI “Fertility control services”) Published Date: 20120101- AND Apply related words; Apply equivalent subjects” | 5 May 2023 | 59 |
| Embase | (preexposure AND prophylaxis:ab,ti OR 'pre-exposure prophylaxis':ab,ti OR 'pre exposure prophylaxis':ab,ti OR prep:ab,ti) AND hiv:ab,ti AND ('family planning services':ab,ti OR 'family planning service':ab,ti OR 'family planning':ab,ti OR 'planned pregnancy':ab,ti OR 'planned pregnancies':ab,ti OR 'family planning programs':ab,ti OR 'family planning program':ab,ti OR 'family planning programmes':ab,ti OR 'family planning programme':ab,ti OR 'reproductive counselling':ab,ti OR 'birth spacing':ab,ti OR 'birth control':ab,ti OR 'contraception':ab,ti OR 'fertility control':ab,ti OR 'family planning center':ab,ti OR 'family planning centers':ab,ti OR 'family planning centre':ab,ti OR 'family planning centres':ab,ti OR 'family planning clinic':ab,ti OR 'family planning clinics':ab,ti OR 'abortion clinic':ab,ti OR 'abortion clinics':ab,ti OR 'abortion center':ab,ti OR 'abortion centers':ab,ti OR 'abortion centre':ab,ti OR 'abortion centres':ab,ti OR 'contraceptive services':ab,ti OR 'reproductive health services':ab,ti OR 'reproductive health service':ab,ti OR 'maternal health services':ab,ti OR 'sexual and reproductive health service':ab,ti OR 'sexual and reproductive health services':ab,ti OR 'fertility control clinics':ab,ti OR 'fertility control clinic':ab,ti OR 'fertility control center':ab,ti OR 'fertility control centers':ab,ti OR 'fertility control centre':ab,ti OR 'fertility control centres':ab,ti OR 'fertility control services':ab,ti) AND [2012-2023]/py | 5 May 2023 | 274 |
| Sociological Abstracts | noft(("Family Planning services" OR "Family Planning service" OR "Family Planning" OR "Planned Pregnancy" OR "Planned Pregnancies" OR "Family Planning Programs" OR "Family Planning Program" OR "Family Planning Programmes" OR "Family Planning Programme" OR "Reproductive counselling" OR "Birth Spacing" OR "Birth Control" OR "Contraception" OR "Fertility control" OR "family planning center" OR "family planning centers" OR "family planning centre" OR "family planning centres" OR "Family planning clinic" OR "Family Planning Clinics" OR "Abortion clinic" OR "Abortion clinics" OR "Abortion center" OR "Abortion centers" OR "Abortion centre" OR "Abortion centres" OR "Contraceptive services" OR "Reproductive health services" OR "Reproductive health service" OR "Maternal health services" OR "Sexual and Reproductive health service" OR "Sexual and Reproductive health services" OR "Fertility control clinics" OR "Fertility control clinic" OR "Fertility control center" OR "Fertility control centers" OR "Fertility control centre" OR "Fertility control centres" OR "Fertility control services")) AND noft(HIV) AND noft((pre-exposure prophylaxis OR "preexposure prophylaxis" OR "pre-exposure prophylaxis" OR "pre exposure prophylaxis" OR PrEP)) AND pd(>20120101) | 5 May 2023 | 10 |
